# Supplementary material for: Identification of photoperiod-induced specific miRNAs in the adrenal glands of Sunite sheep (Ovis aries)
Source: Front Vet Sci. 2022 Jul 22;9:888207. doi: 10.3389/fvets.2022.888207 (PMC9354845; doi:10.3389/fvets.2022.888207)
Supplement: Supplementary file 1 [file Data_Sheet_1.ZIP › Supplementary file/miRDeep2 file.zip!/miRDeep2 file/Know_Structure/SPLP42A3/oar-mir-299.pdf]

miRBase precursor : oar-mir-299
Total read count : 151
oar-miR-299-5p read count : 143
oar-miR-299-3p read count : 8
remaining reads : 0

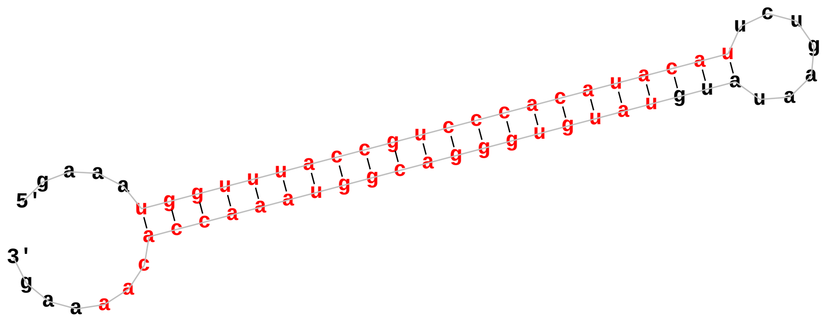

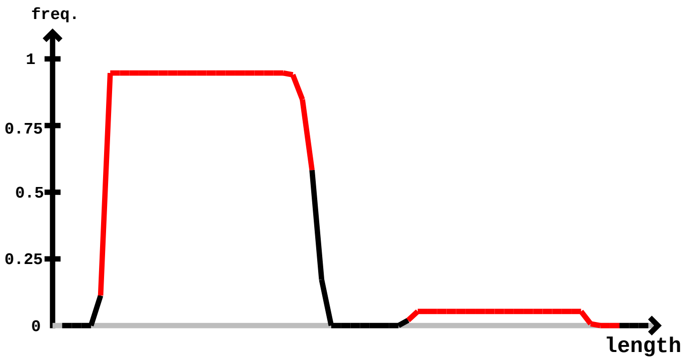


```
        oar-miR-299-5p
                                    oar-miR-299-3p
5'- gaaaugguuuaccgucccacauacauucugaauauguaugugggacgguaaaccacaaaag   -3'    exp
    ....((((((((((((((((((((((.......))))))))))))))))))))))......    reads     mm        sample
    ...augguuuaccgucccacaua......................................    1         0         seq
    ...augguuuaccgucccacauac.....................................    2         0         seq
    ...augguuuaccgucccacauaca....................................    14        0         seq
    ....ugguuuaccgucccacauac.....................................    12        0         seq
    ....ugguuuaccgucccacauaca....................................    26        0         seq
    ....ugguuuaccgucccacauacau...................................    62        0         seq
    ....ugguuuaccgucccacauacauu..................................    14        0         seq
    ....ugguuuaccgucccacauacauA..................................    12        1         seq
    ...................................guaugugggacgguaaacc.......    2         0         seq
    ...................................guaugugggacgguaaacca......    1         0         seq
    ....................................uaugugggacgguaaacc.......    5         0         seq
```
